# Horizontal Directional Drilling-Length Detection Technology While Drilling Based on Bi-Electro-Magnetic Sensing

**DOI:** 10.3390/s17050967

**Published:** 2017-04-27

**Authors:** Yudan Wang, Guojun Wen, Han Chen

**Affiliations:** School of Mechanical and Electronic Information, China University of Geosciences, Wuhan 430074, China; wangyodan@163.com (Y.W.); chenhan_cug@163.com (H.C.)

**Keywords:** horizontal directional drilling, real-time drilling-length detection, bi-electromagnetic sensing, magnetic-field strength changes, LabVIEW

## Abstract

The drilling length is an important parameter in the process of horizontal directional drilling (HDD) exploration and recovery, but there has been a lack of accurate, automatically obtained statistics regarding this parameter. Herein, a technique for real-time HDD length detection and a management system based on the electromagnetic detection method with a microprocessor and two magnetoresistive sensors employing the software LabVIEW are proposed. The basic principle is to detect the change in the magnetic-field strength near a current coil while the drill stem and drill-stem joint successively pass through the current coil forward or backward. The detection system consists of a hardware subsystem and a software subsystem. The hardware subsystem employs a single-chip microprocessor as the main controller. A current coil is installed in front of the clamping unit, and two magneto resistive sensors are installed on the sides of the coil symmetrically and perpendicular to the direction of movement of the drill pipe. Their responses are used to judge whether the drill-stem joint is passing through the clamping unit; then, the order of their responses is used to judge the movement direction. The software subsystem is composed of a visual software running on the host computer and a software running in the slave microprocessor. The host-computer software processes, displays, and saves the drilling-length data, whereas the slave microprocessor software operates the hardware system. A combined test demonstrated the feasibility of the entire drilling-length detection system.

## 1. Introduction

Horizontal directional drilling (HDD) technology is widely used in underground pipeline installation and horizontal oil and gas wells to drill horizontal boreholes. HDD technology (also called trenchless technology) is the most popular method for underground pipeline construction because it causes little damage to the ground, little disturbance to the surrounding residents, has a high efficiency, and is low-cost. HDD technology is also indispensable for the efficient collection of oil and gas, particularly for unconventional gas in low-permeability gas reservoirs, such as coal bed methane [1,2].

The drilling length is a significant parameter in horizontal borehole path design and drilling-state monitoring. It can be used for fitting the drilling profile, computing the deviation, designing the drilling profile, and developing an algorithm for automated directional drilling [3]. In addition, the length of the horizontal well has a considerable impact on the oil and gas production [4]. Scientific planning of the drilling length has practical significance for controlling the horizontal well trajectory and reducing the exploration cost for the drilling of horizontal boreholes. Data regarding the drilling length constitute basic information for making decisions during the drilling of horizontal boreholes.

Accurate horizontal drilling-length data is essential for making correct judgments and decisions during drilling and has thus been increasingly emphasized by many oil and production units and administrative departments. Only by accurately measuring and formally managing the drilling length, can the distribution and situation of the drilling borehole be understood for safe production and efficient management.

Although the drilling length is very important, accurate statistics regarding it are lacking, as there is no efficient and scientific method for accurately measuring the drilling length for each borehole in real time during drilling.

Regarding the drilling length of vertical boreholes, only some of the multi-parameter drilling apparatuses developed by the enterprises and universities can measure the drilling length in real time [5,6,7,8,9]. They detect the drilling length according to the hook load, the encoders fixed on the winch, and the casing collar. Not all of these devices are available for horizontal drilling. Furthermore, it is impossible to measure the drilling length for horizontal boreholes in deep stratum after the drilling, owing to the shrinkage and local collapse of the boreholes.

Presently, the only simple and feasible way to measure the drilling length of horizontal boreholes in the deep stratum is to count the number of drill stems artificially. The artificial counting of drill items is monotonous and boring; moreover, it is prone to error with a long work time. Inaccurate data regarding the drilling length inevitably lead to erroneous judgments and decisions, which may result in safety accidents.

Directional drilling technology has gradually been adopted by the pipe-installation and oil & gas industries, the display device of the guider cannot directly determine the drilling length via either measuring while drilling (MWD)—which is a mature technology—or logging while drilling (LWD), which has gained popularity in recent years. The drilling length can be calculated by determining the azimuth and inclination of the horizontal borehole and measuring the vertical buried depths beneath the ground surface using electromagnetic waves [10,11]. However, this method is only suitable for shallow boreholes, e.g., those used for pipeline installation. It is not applicable to deep strata hundreds or thousands of meters below the ground surface, because of the uncertain attenuation characteristics of the electromagnetic waves in various formations.

As described in a patent called “Drilling Length Detection Method and Measuring Instruments for Gas Drainage Borehole in Coal Mine”, the horizontal drilling length can be determined by measuring the hydraulic oil flow of the hydraulic cylinder [12]. This indirect measurement method may be effective in ideal working conditions. However, it ignores the differences between the ideal situation and reality and underestimates the degree of difficulty of cracking various rocks, the volume loss of the hydraulic system, the decrease in the quality of the hydraulic oil due to the pollution in the hydraulic cylinder, the temperature drift of the hydraulic oil, and so on. Furthermore, this method is invalid for drilling rigs that are not driven by hydraulic motors, such as rigs driven by rack and pinion gears.

The measurement of drilling length has received attention in recent years. More recently, there have been researches about the measurement methods. The most popular method is to apply stress wave or sonic wave to determine the drilling length by detecting the wave travel time and phase feature in the drill string [13,14]. To avoid the disturbance from the vibration of the drill strings while drilling, the drill rig has to stop working so that the detector can receive the sonic signal from knocking on the drill strings. Furthermore, due to serious signal attenuation of sonic wave in the drill strings, the drilling length to be measured is limited to a few dozens of meters [15,16]. A low-voltage pulse is applied to the drill stem with cable, to measure the length of drill string by detecting the travel time of pulse signal in cable [17], but the signal attenuation is serious owing to the mud in drill stems. The signal is not stable and reliable with a long drill string [18]. A method for counting drill stems with monitor video of drill rig was proposed in 2015 [19]. The number of drill stems is counted by technology of machine vision. To facilitate the images processing and analysis, the monitor video must be recorded in strict environment, which is difficult to be achieved in the site. The accuracy of measurement results relies on the images quality. Many factors including illumination changes, target occlusion, shadow interference, etc., will affect the images quality, thus possibly leading to miscounting the number of drill stems.

In accordance with the aforementioned points, most existing multi-parameter detectors are used for vertical wells, whereas the patented drilling-length detector for measuring the hydraulic oil flow is only applicable under strictly ideal conditions. Currently, there is no available instrument for the length detection of horizontal drilling, especially in deep strata. This is because of the complex drilling process: frequent rechucks occur, along with the round trip of the drill stems and switching within strings during drilling, making it difficult to clarify the process of horizontal drilling automatically. In addition, although the drilling length is far more significant for sub-horizontal drilling than for all other types of directional drilling, directional-drilling experts and researchers direct their efforts toward mud logging while drilling, logging while drilling, employing seismic technology while drilling, and employing down hole pressure-monitoring systems while drilling and thus ignore the most basic drilling parameter: the drilling length. In recent years, there are some researches about this parameter, but all the methods have some limitations.

This paper presents a contactless and direct detection method based on bi-electromagnetic sensing for acquiring the horizontal drilling-length data. In this method, the data are obtained automatically instead of via manual counting. Compared with indirect and contact detection, the proposed method can extend the service life of sensors, significantly improve the accuracy of drilling, and ensure the speed of drilling. The utilization of the proposed detection device and software for measuring the drilling length during drilling will greatly improve the capabilities of drilling rigs and drilling technology. This can reduce the labor intensity for workers, prevent human errors in the measurement and recording of the drilling length, and make it easy to statistically analyze the drilling length. Thus, the proposed method can significantly contribute to correct judgments and decisions under various drilling conditions.

## 2. Basic Mechanism of Drilling-Length Detection Based on Bi-Electromagnetic Sensing

### 2.1. Basic Computation Principle for Drilling Length

The basic computation principle is similar to that of manual counting by multiplying the length of each drill stem by the number of drill stems. The length of each drill stem is a standard value, and the number of drill stems is determined by the number times that the joints of the drill stems pass through the clamp, inward and outward. Given that the real-time drilling length is *L*, the length of a drill stem is *l*, the number of forward joints of the drill stem is *n*_1_, and the number of backward joints of the drill stem is *n*_2_, the drilling length is determined using Equations (1) and (2).

If the initial drilling length is 0, the real-time drilling length is:

(1) $$L=l\times (n_{1}-n_{2})$$

If the initial drilling length is not 0 but *l*_0_, the real-time drilling length is:

(2) $$L=l_{0}+l\times (n_{1}-n_{2})$$

### 2.2. Optimized Detection Method

**Figure 1:**
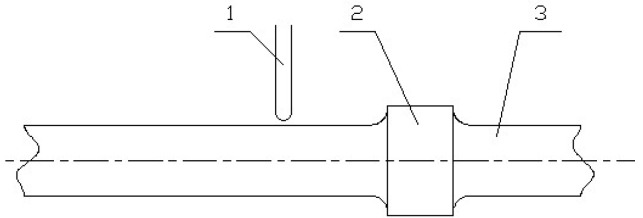
Schematic of contact-type detection: 1—contact probe; 2—drill-stem joint; 3—drill stem.

Two parameters—*n*_1_ and *n*_2_—are necessary for the calculation of the drilling length. There are two methods for obtaining *n*_1_ and *n*_2_. One is a contact-type method, and the other is contactless. The contact-type detection method utilizes a contact-type probe to identify the difference in outer diameter between the drill stem and the drill-stem joint for the internal flush drill stem. This difference must be increased via artificial reconstruction for the external flat drill stem. When the drill stem and drill-stem joint move, the contact probe does not touch the drill stem but scratches the drill-stem joint, as illustrated in Figure 1.

**Figure 2:**
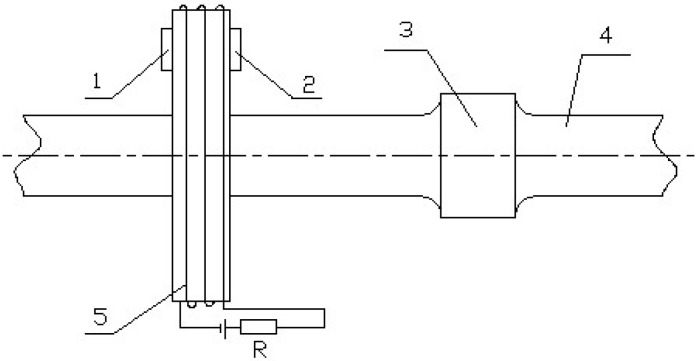
Schematic of contactless detection: 1—magnetoresistive sensor A; 2—magnetoresistive sensor B; 3—drill-stem joint; 4—drill stem; 5—current coil.

The contactless detection method utilizes an electromagnetic effect to identify the difference in the structure or wall thickness between the drill stem and drill-stem joint for either the internal flush drill stem or the external flat drill stem. When the drill stem or drill-stem joint passes through the current coil, the magnetic field produced by the current coil is changed differently [20], as illustrated in Figure 2.

The contact-type detection method has the advantages of a simpler device and lower initial cost than the contactless method; however, it requires a high reliability for the contact probe and is only suitable for the internal flush drill stem, whose outer diameter becomes larger at the joint. For the external flat drill stem without artificial reconstruction, whose outer diameter remains constant at the joint, this method fails.

In comparison, the contactless detection method has a complex device and a higher initial cost but a longer lifetime and higher reliability, as there is no friction between the detecting device and the drill stems. Additionally, the results are not affected by drilling cuttings attached to the surface of the drill stems during the drilling. Thus, the contactless detection method is the ideal choice for detecting the horizontal drilling length.

### 2.3. Theoretical Basis for Computing Magnetic-Field Strength

**Figure 3:**
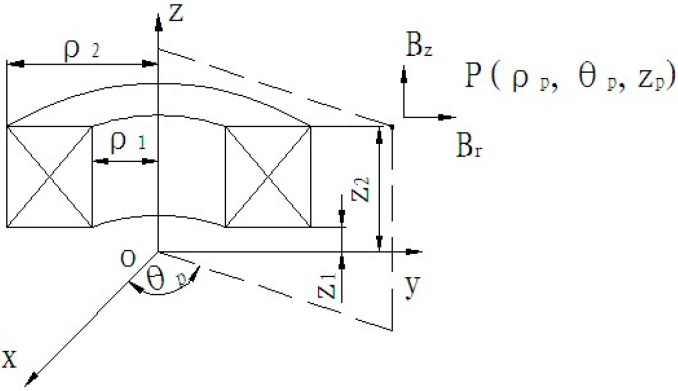
Model for computing the field strength.

The current coil for contactless detection can be considered as a solenoid, as illustrated in Figure 3. The magnetic-field strength at an arbitrary point (P) around the solenoid can be calculated using Equations (3)–(6) [21].

(3) $$B_{\rho }=\frac{\mu_{r}\mu_{0}J}{4\pi }\int_{-\pi }^{\pi }d\theta \int_{\rho_{1}}^{\rho_{2}}d\rho \int_{z_{1}}^{z_{2}}\frac{-\left(z-z_{P}\right)\rho \cos\theta }{r^{3}}dz$$

(4) $$B_{z}=\frac{\mu_{r}\mu_{0}J}{4\pi }\int_{-\pi }^{\pi }d\theta \int_{\rho_{1}}^{\rho_{2}}d\rho \int_{z_{1}}^{z_{2}}\frac{\rho \left(\rho -\rho_{P}\cos\theta \right)}{r^{3}}dz$$

(5) $$r={\left[\rho^{2}+\rho_{P}^{2}-2\rho \rho_{P}\cos\theta +{\left(z-z_{P}\right)}^{2}\right]}^{1/2}$$

(6) $$J=\frac{nI}{\left(\rho_{2}-\rho_{1}\right)\left(z_{2}-z_{1}\right)}=\frac{nU}{\left(\rho_{2}-\rho_{1}\right)\left(z_{2}-z_{1}\right)\left(R1+R2\right)}$$

Here, *B_z_* is the axial magnetic-field strength at point P; *B_ρ_* is the radial magnetic-field strength; *μ_r_* is the relative magnetic permeability; *μ*_0_ is the permeability of vacuum; *ρ*_1_ is the inner radius of the solenoid coil; *ρ*_2_ is the external radius of the solenoid coil; *θ_P_*, *ρ_P_* and *z_P_* are the cylindrical coordinates of point P; *J* is the current density; *z*_1_ is the axial coordinate of the bottom plane of the solenoid coil; *z*_2_ is the radial coordinate of the upper surface of the solenoid coil; *n* is the number of turns of the electromagnetic coil; *U* is the service voltage; *R*1 is the resistance of the electromagnetic coil; *R*2 is the coil resistance.

### 2.4. Distinguishing Drill-Stem Joint from Drill Stem

Many materials—including air, the drill stem, and mud—pass through the current coil during the drilling process. Changes in these materials affect the relative dielectric constant *μ_r_*, thereby altering the strength of the magnetic field around the coil. The most significant factor is the drill stem, which is made of a ferromagnetic material, because the magnetic field is extremely sensitive to the difference in the cross-section between the drill stem and the drill-stem joint. Thus, *n*_1_ and *n*_2_ can be determined by detecting the change of the magnetic-field strength that occurs when the drill stem and drill-stem joint successively pass the current coil.

### 2.5. Judgement of Movement Direction

The movement direction of the drill stem must be identified according to the order of the responses of Sensors A and B. The magnetic-field changes that occur when the drill stem and drill-stem joint perform forward or backward movement have the same direction, even if their magnitudes differ. If Sensor A indicates the change of the magnetic field prior to Sensor B, the power unit is in the status of feeding in the drill stem, i.e., performing forward movement; thus, the number of forward drill-stem joints *n*_1_ is increased by 1 and written to the memory chip in terms of the real-time change. Otherwise, the power unit is in the status of hoisting the drill bit, i.e., performing backward movement; thus, the number of backward drill-stem joints *n*_2_ is increased by 1 and then written to the memory chip in terms of the real-time change.

### 2.6. Data acquisition and Processing

The detection is continuously performed while the HDD drilling rig is working. During the detection, the acquired data are stored in the memory cell of slave computer and the operator of drilling rig can directly observe the measurement results displayed on the small screen of slave computer. For further data management, the data from detection system of each HDD drilling rig are transferred to the drilling-length data-management system (developed using the software LabVIEW; National Instruments, Austin, TX, USA) in the host computer.

The slave computer works on the spot while the host computer with data-management system works at the administration center. If the slave computer is on the ground as the host computer, the data between them can be transferred by either wireless transmission or portable storage unit like USB (universal serials bus) drive.

However, as the HDD drilling in the coal seam is at least 100 m beneath the ground, the data transmission is difficult by wireless way. The detection system setup on the HDD drilling rig is available for the operator on the spot, not for the administrators on the ground. Therefore, the memory cell (USB drive) full of data will be carried to the ground after a day work in coal seam.

## 3. Design of Contactless Detection System Based on Bi-Electromagnetic Sensing

### 3.1. General Scheme Design

**Figure 4:**
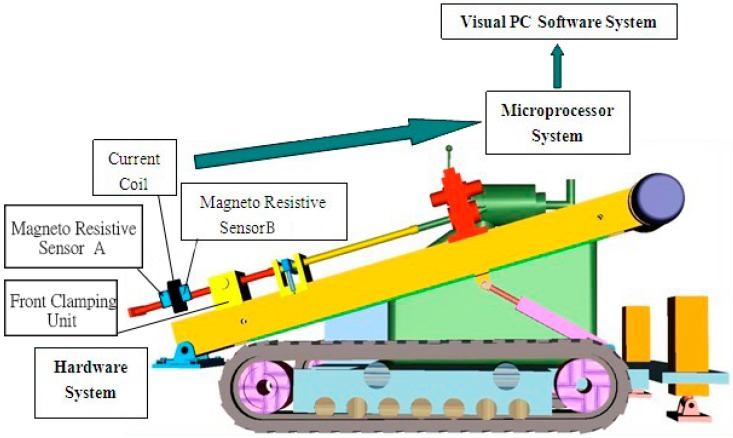
Installation diagram of contactless detection system.

According to the aforementioned analysis of the drilling-length detection based on double electromagnetic sensing, an installation diagram of the contactless detection system is presented in Figure 4.

The current coil is fixed beside the front clamping unit, coaxially with the drill stem. Two magnetoresistive sensors—Sensors A and B—are fixed on the sides of the current coil symmetrically. They detect the real-time different changes of the magnetic field produced by the movement of the drill stem and the drill-stem joint through the current coils. A critical value is set to judge whether the drill-stem joint or drill-stem is passing through the clamping unit.

**Figure 5:**
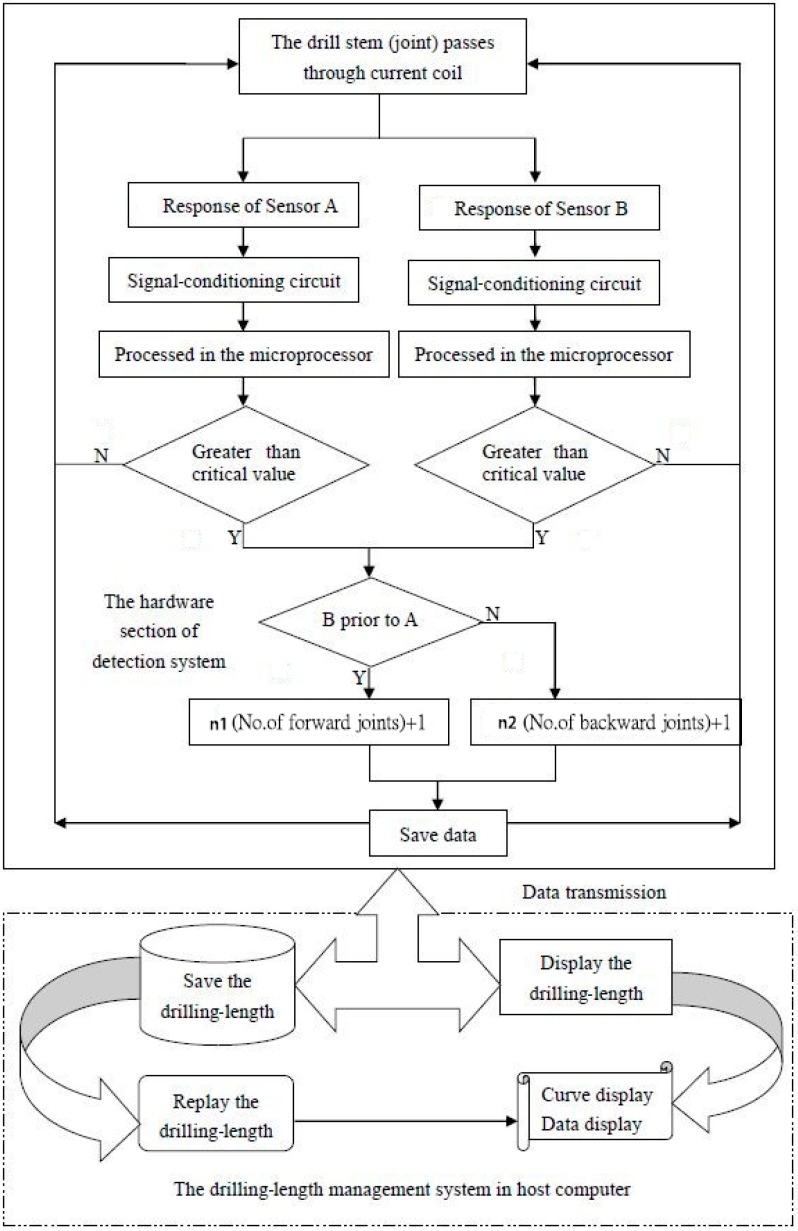
Flow chart of contactless detection.

According to the aforementioned general scheme, the working flow of the contactless detection system is illustrated in Figure 5.

### 3.2. Design for Microprocessor System

**Figure 6:**
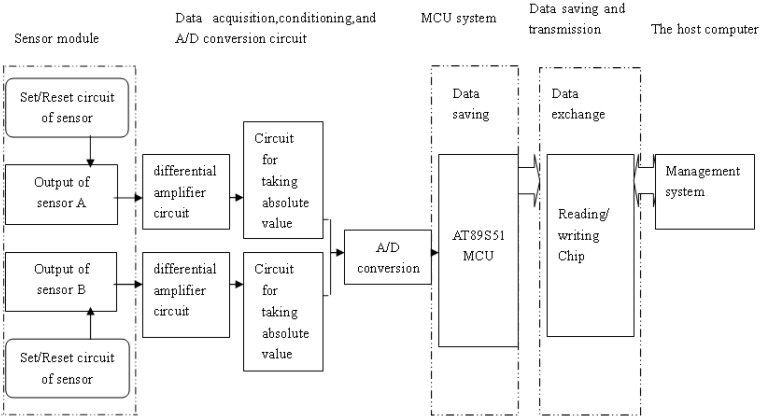
Hardware structure of the horizontal drilling-length detection system.

A structural diagram of the microprocessor system for the drilling-length detection system for directional drilling is illustrated in Figure 6. The detection system collects real-time signals from the two magneto resistive sensors. After passing through a series of signal-conditioning circuits for differential amplification, filtering, taking the absolute value, and analog-to-digital (A/D) conversion, the signals of the magneto resistive sensors are transmitted to the micro programmed control unit (MCU). The signals are processed by the MCU to determine whether the drill-stem joint passes through the current coil and determine the movement direction according to the responding sequence of the two magneto resistive sensors. Then, the drilling-length data processed by the MCU are stored in the memory chip as digital data.

### 3.3. Software for Contactless Electromagnetic Detection System

The system software is composed of the software in the host computer and the software in the slave computer. The visual host-computer software processes, displays, and saves the drilling length, and the slave-computer software drives all the operations of the hardware system.

#### 3.3.1. Slave-Computer Software

The slave-computer software consists of a sensor-data acquisition module, a module for data transmission between the host and slave computers, and an inter-integrated circuit (I^2^C) communication module.

**Figure 7:**
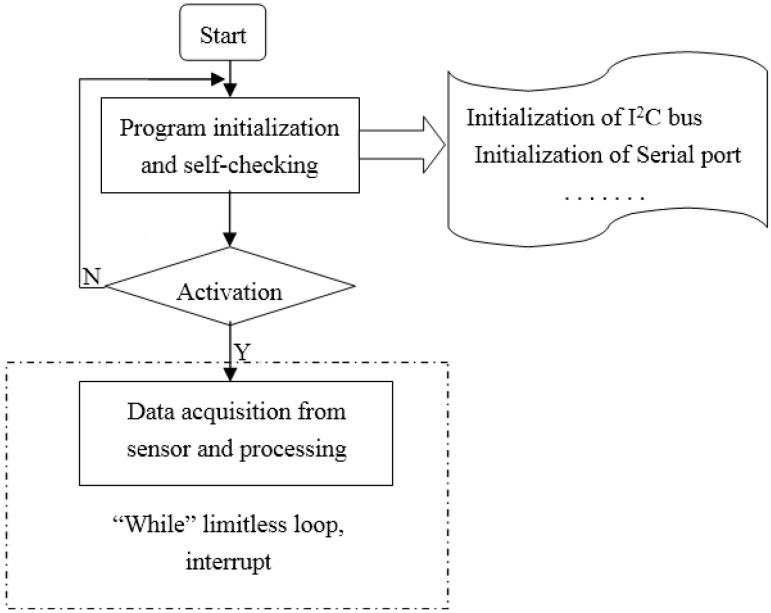
Flow chart of the main program.

The slave computer programming design can be divided into five programs: the sensor data acquisition and processing subprogram, I^2^C communication subprogram, interrupt subprogram, subprogram for data transmission between the slave and host computer subprograms, and main program. The main program performs the data acquisition, processing, and saving by calling the corresponding subprograms. A flow chart of the main program is shown in Figure 7. Flow charts of the subprograms are not shown herein.

#### 3.3.2. Host-Computer Software

The host-computer software of the drilling-length management system is compiled via the programming methodology of Virtual Instrument Software Architecture in a LabVIEW environment [22]. All the performances are realized in four optional visual tabs: (1) login; (2) serial communication; (3) data processing, display, and storage; and (4) replay for historical data.

##### Login

**Figure 8:**
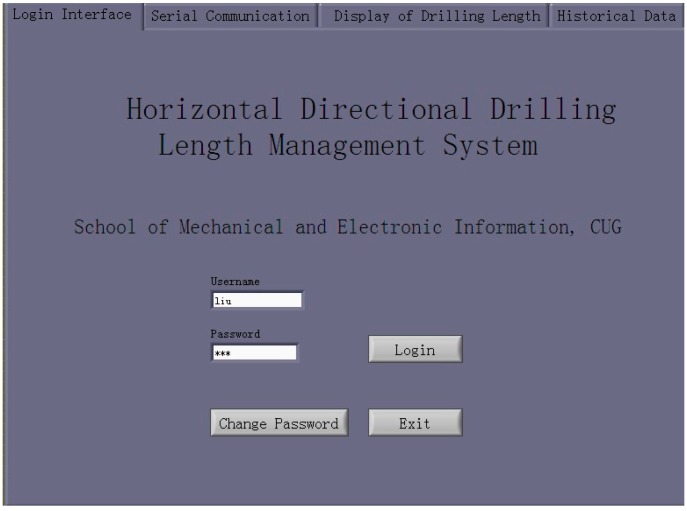
Login interface.

The login tab is a friendly welcome interface with three functions: (1) allowing the user to input the username and password; (2) switching to the serial-communication tab automatically when a correct username and password are entered; and (3) allowing the user to modify the password. The login interface is shown in Figure 8.

##### Serial Communication

The serial-communication interface is used for data transmission between the slave computer and host computer. It consists of seven modules with the following functions: (1) initialization; (2) writing data; (3) reading data; (4) clearing data; (5) initializing the serial ports; (6) displaying data while receiving; and (7) sending data.

The data transmission employs the serial-communication protocol, as follows:

When the system starts, the host computer (personal computer), monitors the COM ports. When it is connected to the slave computer, the system sends a command string requesting data transmission. This command string consists of a starting character, a command code, and an ending character. The command code is preset as “S”.

When the slave computer receives this command string, it immediately tests and verifies the string. If the string is valid, the slave computer sends the data in the format “#A/Bn*”, where # is the starting character, A indicates forward motion, B indicates backward motion, n is the amounts of forward or backward motion, and * is the ending character and the storage command. If the string is not valid, the slave computer continues to wait for the correct command string. 

After the host computer receives all the required data, it identifies the first character of the data. If this character is “#”, the data are processed and displayed. Then, the host computer identifies the fourth character. If this character is “*”, the data and time (after being processed) are stored in an Excel file automatically produced by the management system.

**Figure 9:**
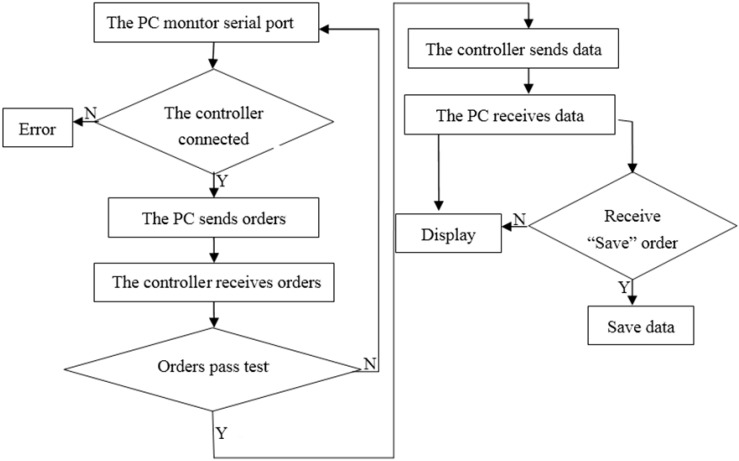
Flow chart of the data communication between the slave and host computers.

A flow chart of the data communication between the slave and host computers is shown in Figure 9.

**Figure 10:**
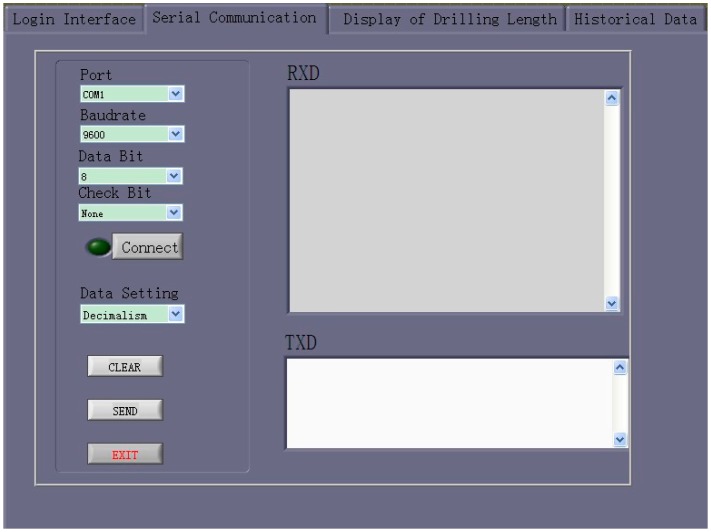
Serial-communication interface of the visual drilling-length management software.

The serial-communication interface of the visual drilling-length management software is shown in Figure 10.

##### Data Processing, Display, and Storage

When the host computer receives the character “#”, it identifies the second character. If this character is “A”—indicating forward motion—the third character is converted into a numerical value, which is added to the number of forward joints of the drill stem. Alternatively, if it is “B”—indicating backward motion—the third character is converted into a numerical value and added to the number of backward joints of the drill stem.

Then, the updated drilling length is displayed as two types of figures and a graph. Additionally, the maximum data computed using Equations (1) or (2) are displayed.

**Figure 11:**
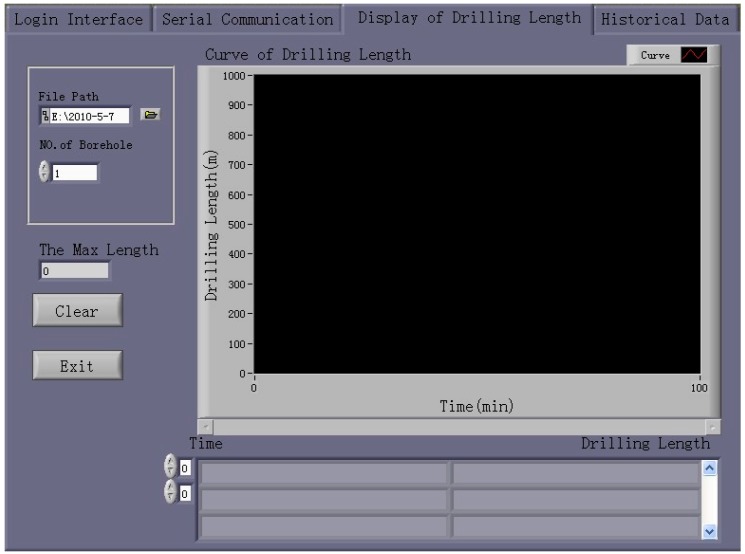
Data display and storage interface of the visual drilling-length management software.

Finally, the computer receives the fourth character—“*”—the drilling length and corresponding time are stored in an Excel file whose filename indicates the date and the number of boreholes. The storage directory can be set by the user. The interface for displaying and storing data in the visual drilling-length management software is shown in Figure 11.

##### Replay for Historical Data

The system allows the user to view data stored previously, which are represented as two types of figures and a graph. The user can select a historical data file using the “Open File Path” dialog and then click the button labeled “Replay for Historical Data” to view the historical data.

## 4. Combined Tests and Discussion

After the hardware system was constructed and the software system was debugged, combined tests were performed to evaluate the feasibility and performance of the method.

### 4.1. Manufacture and Testing of Current Coil

After more than 10 experiments, a copper wire with a diameter of 0.35 mm was wound 3000 times around a plastic tube with an outer diameter of 110 mm to form the coil. Two magnetoresistive sensors were placed at the sides of the coil. Then, the coil was connected to a 5-V supply. The parameters of Equations (3) and (4) were shown in Table 1.

Accordingly, the axial magnetic-field strength and radial magnetic-field strength at point P were determined as as *B_ρ_* = 1.5 Gs and *B_z_*= 0.1 Gs respectively, for the case where neither the drill stem nor drill-stem joint passed through the current coil. Because *B_z_* was significantly smaller than *B_ρ_*, it was ignored. During the test, the output average strength of magnetic-field around the current coil measured at point P was approximately 1.51 Gs, which confirms the validity of the experimental method and the current coil.

### 4.2. Manufacture of Circuit Boards

Printed circuit boards (PCBs) were fabricated according to the hardware scheme. Considering the limitations of the PCB shape and the internal dimensions of the clamping units, the circuit boards for the sensor module and the microprocessor module were made and placed separately according to the circuits of the hardware system. The sizes of the circuit boards for the sensor and microprocessor modules were 32 mm × 65 mm and 32 mm × 200 mm, respectively.

### 4.3. Installation of Hardware System

**Figure 12:**
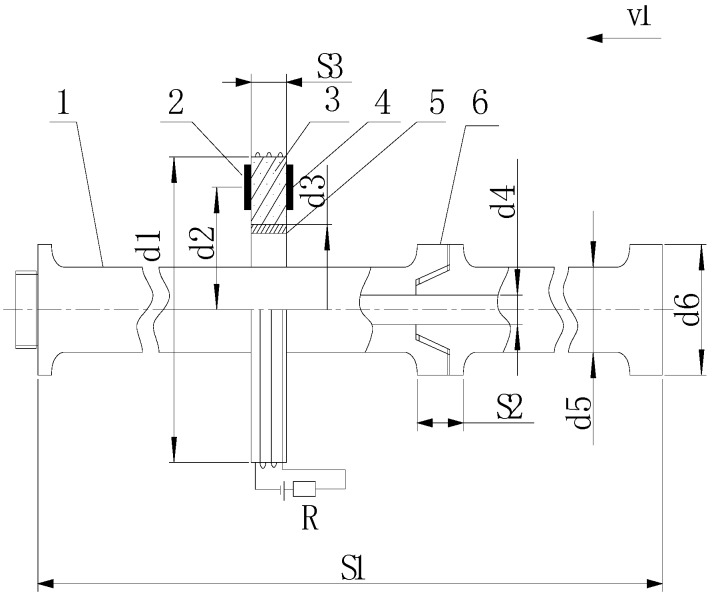
Installation of the detection device: 1—drill stem; 2—magnetoresistive sensor B; 3—current coil; 4—magnetoresistive sensor A; 5—plastic tube; 6—drill-stem joint.

A ZT-18 HDD rig with drill stems 76 mm in diameter and 3 m in length was employed to perform the drilling process according to the installation diagram of the contactless detection system, which was previously shown. The detailed installation of the detection device is illustrated in Figure 12.

Here, S1 is the total length of the two connected drill stems (6 m), S2 is the length of the drill-stem joint (200 mm), d5 is the outer diameter of the drill stem (76 mm), d4 is the inner diameter of the drill stem (46 mm), d6 is the outer diameter of the drill-stem joint (86 mm), d3 is the diameter of the plastic tube (110 mm), S3 is the width of the plastic tube around which the current coil was wound (30 mm), d1 is the outer diameter of the current coil (150 mm), and d2 is the distance between the center of the coil and the magneto resistive sensor (70 mm).

### 4.4. Test for Difference between Drill Stem and Drill-Stem Joint

**Figure 13:**
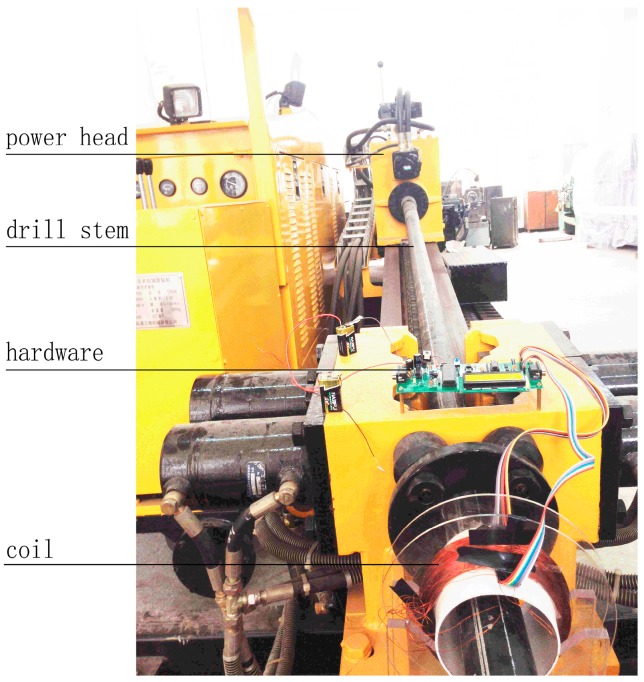
Experiment for measuring the magnetic-field strength.

The directional drilling rig ZT-18 was employed to perform experiments. The sensor applied in the experiments was HMC1021Z magneto resistive sensor made by the Honeywell Company (Morristown, NJ, USA) with a detection range of −6 to +6 Gs. The drill stem and drill-stem joint moved forward in turn. The experiment is illustrated in Figure 13.

**Figure 14:**
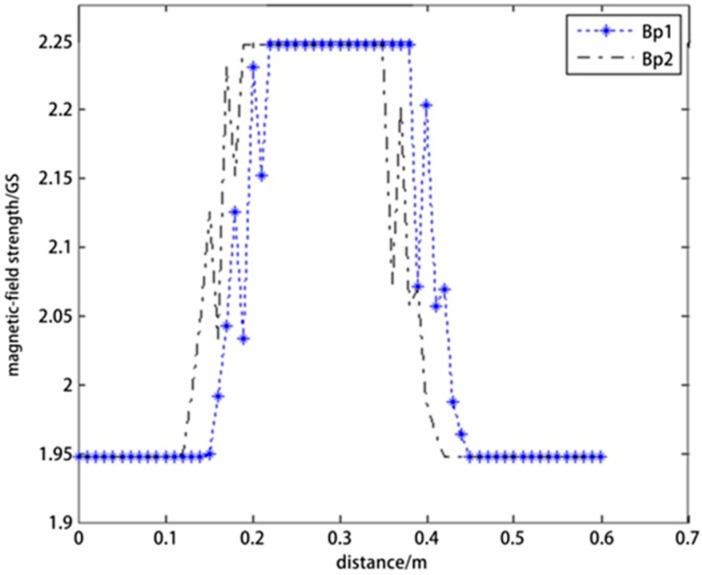
Magnetic-field strength measured by magneto resistive sensors A and B.

The square waves of the output voltages measured by two magneto resistive sensors are illustrated in Figure 14, which shows that the output voltage remained low when the drill stem passed through the current coil and increased to a high level when the drill-stem joint passed through the current coil.

*B_ρ_*_1_ is the value detected by magneto resistive sensor A, and *B_ρ_*_2_ is the value detected by magneto resistive sensor B. When the drilling stem was in the current coil, *B_ρ_* was approximately 1.9 Gs, and when the drilling stem joint was in the current coil, *B_ρ_* was approximately 2.2 Gs.

Many tests revealed that the magnetic-field strength was consistent and smooth when the drill stem passed through the current coil. However, when the joint passed through the coil, there was a sudden increase, and then the magnetic-field strength remained at a high level. After the joint had completely passed through, the magnetic-field strength returned to the normal level. Many tests also revealed that the cross section of drill string is the most important factors affecting the differential value of magnetic field strength around sensor A and B. The possible problems of installation, such as the asymmetry of the two sensors, the coaxially error between the current coil and drill stem, will not affect the differential value.

### 4.5. Display and Redisplay of Test Results

**Figure 15:**
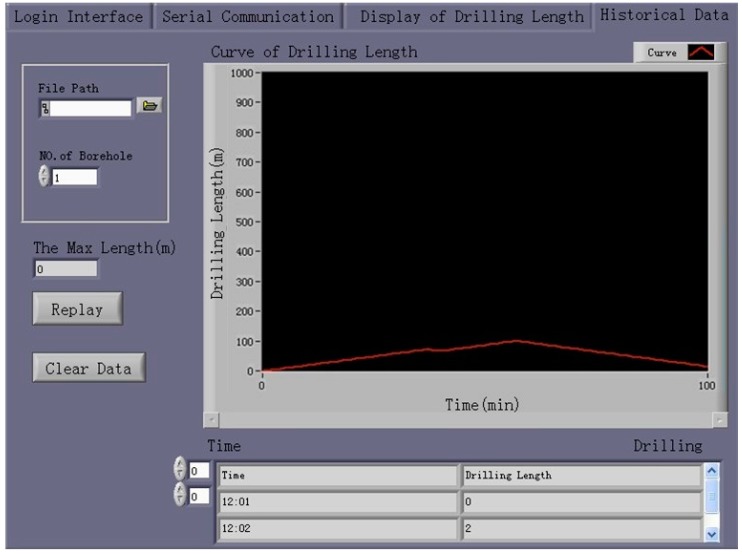
Replaying of the drilling-length results obtained using the visual drilling-length management software.

During the combined test, the response of the drilling-length management system running in the host computer was observed in real time. After the test, the test results were replayed using the visual drilling-length management software, as shown in Figure 15.

### 4.6. Discussion

To assess potential limitations of the measurements, it is necessary to understand the influence of environmental factors. According to the differential value measured by the two sensors, the moving direction of drill stem is identified and the number of drill stem is counted. The axial-transverse section of drill string is the most significant factors affecting the differential value of magnetic field strength. The other factors, temperature, vibrations, pipe entry angle and so on, may affect the magnetic field intensity, but will not change the differential value of magnetic field strength when the drill stem and drill stem joint passes through the coil. Therefore, the measurement results are not affected by environmental factors.

Another possible concern for the detections could be the applicability of this electromagnetic sensing technique. Obviously, it is effective for ferromagnetic material, which is most widely used in the manufacture of drill stem. Therefore, this technique can be applied in almost all HDD. Additionally, in the experiments, the inner diameter of coil circle is 110 mm, so it can be applied to the drill stem with outer diameter less than 110 mm. Theoretically, this technology can be applied to drill stem with various diameter based on the inner diameter of coil circle. The sensors are configured as a 4-element Wheatstone bridge. These highly sensitive sensors are capable of sensing magnetic fields as low as 30 µGs with operating temperature of −40 to 85 °C, which can meet the demands of environment and working condition. 

## 5. Conclusions

This paper presents a new method for the real-time detection of the HDD length and a management system based on bi-electromagnetic sensing using a microprocessor, two magneto resistive sensors, and the software LabVIEW. After a detailed analysis of the principle of electromagnetic detection technology, the hardware and software systems were designed, manufactured, and compiled. Then, combined tests were performed for testing the feasibility of the complete drilling-length detection system. These tests verified that changes in the magnetic-field strength caused by the drill-stem joint passing through the current coil were detected by the corresponding magneto resistive sensor and that the movement direction of the drill stem was identified according to the order of the responses of the two magneto resistive sensors. The tests also showed that both the hardware system and the software system function well and satisfy the requirements of drilling-length detection for HDD.

## Figures and Tables

**Table 1:** Parameter values.

Parameters (Units)	Values
*μ*_0_ (N/A^2^)	4*π* × 10^−7^
*ρ*_1_ (m)	0.055
*ρ*_2_ (m)	0.075
*z*_1_ (m)	−0.015
*z*_2_ (m)	0.015
*z_p_* (m)	0.02
*ρ_p_* (m)	0.07
*U* (V)	5
*R*_1_ (Ω)	275
*R*_2_ (Ω)	330
*n*	3000
*μ_r_*	1
